# Energetics of free and tethered flight in bumblebees (*Bombus terrestris*, Linnaeus 1758)

**DOI:** 10.1098/rsbl.2025.0325

**Published:** 2025-11-12

**Authors:** Emily J. Senior, Peter Tickle, Simon M. Walker, Graham N. Askew

**Affiliations:** ^1^ School of Biomedical Sciences, University of Leeds, Leeds, West Yorkshire, UK

**Keywords:** bumblebee, insect, energetics, flight, efficiency, anaesthesia

## Abstract

Many insect species are reluctant to fly freely in wind tunnels, limiting direct free-flight energetics measurements to just two species. More commonly tethered-flight energetics have been investigated, though the effects of tethering on metabolic rate are unclear. Here, we report mass-specific gross metabolic rate (assessed as the rate of carbon dioxide production; ${\overset{˙}{V}}_{{\text{CO}}_{2}}^{*}$) across a speed range (0–4.1 m s^−1^) in bumblebee (*Bombus terrestris*) workers during tethered and free flight in a closed-circuit wind tunnel. ${\overset{˙}{V}}_{{\text{CO}}_{2}}^{*}$ followed a U-shaped relationship with airspeed during both free (*p* = 0.009) and tethered flight (*p* < 0.001). Bees were anaesthetized with isoflurane during tethering, which had no subsequent effect on their metabolic rate (*p* > 0.05), avoiding issues reported during immobilization with CO_2_ or cold exposure. Tethered ${\overset{˙}{V}}_{{\text{CO}}_{2}}^{*}$ was 45% lower than during free flight (*p* < 0.001), but the minimum power speed and the trajectory of the metabolic power–speed relationship (*p *> 0.8) were similar. Overall flight efficiency ranged from 7.3 to 14.7% and did not vary with airspeed. These findings confirm a U-shaped metabolic power–speed relationship in insects, and suggest that tethered flight may approximate free-flight energetics. However, a shift in the maximum range speed to slower speeds during tethered flight warrants caution against using this variable to predict behaviour.

## 1. Introduction

Flight is one of the most energetically costly behaviours in terms of the rate of metabolic energy expenditure and, as such, flight energetics are central to understanding the morphology and behavioural ecology of insects. Nevertheless, a comprehensive analysis of the metabolic costs of insect forward flight is currently lacking. Organismal metabolic rate during flight in insects largely reflects the metabolic energy use of the flight muscles [1] and, provided that flight muscle efficiency is relatively constant, organismal metabolic rate is expected to be directly proportional to the mechanical power requirements of flight [2]. According to aerodynamic theory, mechanical power varies in a U-shaped relationship with flight speed [3] and, therefore, metabolic power is similarly expected to follow a U-shaped relationship with flight speed, as has been observed in birds [4,5] and bats [6,7]. However, the validity of the assumption of constant flight muscle efficiency is uncertain and direct measurements of flight metabolic rate across a speed range are required to establish whether the metabolic power–speed relationship in insects is also U-shaped.

The energetics of hovering have been extensively studied in free-flying insects [2,8]. However, very few measurements in freely flying insects during forward flight have been made, reflecting the challenges of achieving satisfactory flights within the working section of a wind tunnel, suitable for small animal respirometry. We know of only two free-flight studies of forward flight energetics, however, the nature of the relationship between metabolic rate and speed reported differs. In drone flies (*Eristalis tenax*) a shallow U-shaped relationship was observed [9], whereas in bumblebees (*Bombus* spp.) metabolic power was independent of airspeed [10], although some individuals had a shallow U-shaped power–speed relationship [11]. It is unclear whether the absence of a U-shaped relationship in free-flying bumblebees is an experimental artefact, whether bumblebees employ energy-saving strategies at the lower and higher extremes of the speed range or whether flight muscle efficiency is lower at intermediate flight speeds.

Experimental studies of insect forward flight energetics have largely been confined to tethered animals. In tethered flights in locusts (*Schistocerca gregaria*; [12]) and honeybees (*Apis mellifera*; [13]), metabolic power follows a U-shaped relationship with airspeed, as predicted from aerodynamic theory. However, flight metabolic rate during tethered flight may not reflect that during free flight, since the insect does not need to support its body weight. Where free and tethered ‘hovering’ flight energetics have been compared, free flight is 20–50% higher than tethered flights [13–16]. Potential changes in the curvature of the metabolic power–speed relationship between free and tethered flight, may have important consequences for predictions about insects’ behavioural ecology. Therefore, understanding how tethering influences insect flight energetics compared to free flight is essential.

The principal objective of this study was to quantify metabolic energy expenditure in bumblebees (*Bombus terrestris*, Linnaeus 1758) over a speed range during both free flight and tethered flight to determine the shape of the metabolic power–speed relationship in this species. We hypothesized that metabolic rate during tethered flight would be significantly lower than during free flight across the speed range, but that at any speed, the relative flight cost will be similar between the two flight conditions—i.e. the shape of the metabolic power–speed relationship was expected to be similar under both conditions.

## 2. Methods

### (a) Animals

A *B. terrestris* colony (Koppert UK Ltd, Suffolk, UK) was provided with food (nectar and flower pollen) and water ad libitum. Bumblebees had unrestricted access to a Perspex chamber sufficiently large to enable free flight and foraging between artificial flowers loaded with a sucrose solution (50% w/v).

### (b) Experimental conditions

Four groups of bees were used for the experiments: Group 1—tethered flight; Group 2—free flight; Group 3—free flight following isoflurane exposure; and Group 4—speed variation. Group 1 (*n* = 7): bees were anaesthetized (5% isoflurane in 100% oxygen) to rapidly induce a narcotic state. A metal rod was adhered to the prothorax using cyanoacrylate glue, after which the bee was allowed to recover in room air. Exposure to isoflurane did not exceed 2 min. Full recovery was assumed once a flight response could be initiated in response to an airstream, typically 10 min post-anaesthesia. Following recovery, bees were mounted facing into the airstream within the wind tunnel working section. Group 2 (*n* = 8): individuals were placed in the wind tunnel shortly after capture and free-flight metabolic rate was determined. Group 3 (*n* = 9): free-flight metabolic rate after anaesthesia was quantified to test for metabolic effects of isoflurane. Group 4 (*n* = 5): variation in the bees’ speed was determined during free flight in the working section of the wind tunnel.

Mean body mass was not significantly different between free flight (188 ± 18 mg) and free flight following isoflurane exposure (262 ± 97 mg; *t*(14) = −1.965, *p* = 0.070) and free- and tethered-flight groups (181 ± 40 mg) (*t*(13) = 0.898, *p* = 0.386).

## 3. Wind tunnel and respirometry

Metabolic rate during flight was quantified as the gross rate of carbon dioxide production (${\overset{˙}{V}}_{{\text{CO}}_{2}}$) in a closed-circuit temperature-controlled wind tunnel. The working section of the wind tunnel was the original used in Ellington *et al*.’s study [10] and had internal dimensions: 100 × 105 × 175 mm (width × height × length). The overall, recirculating volume of the wind tunnel was approximately 5 l. Throughout the experiments, a continuous sample of air was drawn from the wind tunnel (200 ml min^−1^) to enable measurement of CO_2_ concentration using a CO_2_ analyser (FoxBox, Sable Systems Inc., Las Vegas, NV, USA), after which the analysed sample was returned to the wind tunnel. Over time, due to the insect’s metabolism, the CO_2_ concentration within the wind tunnel increased. Tunnel CO_2_ concentration was recorded continuously using a data acquisition system at a sample frequency of 200 Hz (LabChart version 8 and PowerLab, ADInstruments, Oxford, UK). The respirometry system was calibrated after each experiment by injecting 0.2 ml of 100% CO_2_ via a hypodermic needle into the wind tunnel gas circuit and determining the change in % CO_2_ corresponding to 0.2 ml CO_2_. The response time of the analyser to the calibration bolus injection was approximately 1.5 s across the range of forward flight speeds used in the experiments. The gross rate of CO_2_ production (${\overset{˙}{V}}_{CO_{2}}$) was calculated from the mean gradient of CO_2_ concentration with respect to time for the flight period and expressed relative to the animal’s body mass to give the body mass-specific gross rate of CO_2_ production (${\overset{˙}{V}}_{{\text{CO}}_{2}}^{*}$; electronic supplementary material, figure S1). Airspeed was determined using a Pitot tube positioned in the centre of the working section, where the bees typically flew and varied between 0 and 4.14 m s^−1^. An ultraviolet light was suspended above the working section and illuminated to stimulate flight. Flight behaviour for each free-flight trial was defined as the period from when the insect took off from the ground until their feet made ground contact, and therefore did not include buzzing or walking behaviours, unlike in the previous bumblebee study by Ellington *et al.* [10]. During free flight, the instantaneous speed of the bumblebee has the potential to differ from the mean air speed since the bumblebee can accelerate and decelerate within the working section. In order to quantify the variation in the bumblebee’s speed, body position during free flight was tracked in a lateral view (*n* = 5) at each measured flight speed, using BlackFly S USB3 camera. The difference between the airspeed and the mean horizontal velocity of the bumblebee was −0.016 ± 0.12 m s^−1^ during hovering, 0.039 ± 0.14 m s^−1^ at 0.7 m s^−1^, 0.15 ± 0.15 m s^−1^ at 1.41 m s^−1^, 0.062 ± 0.13 m s^−1^ at 2.58 m s^−1^, 0.10 ± 0.14 m s^−1^ at 3.37 m s^−1^ and 0.031 ± 0.18 m s^−1^ at 4.14 m s^−1^. Variation in mean horizontal velocity increased with increasing flight speed, but this was considered minimal (electronic supplementary material, figure S2). On average, flight duration was 24 ± 18 s. The temperature of the air in the working section of the wind tunnel was maintained at 20.4 ± 0.8°C by passing air through a heat exchanger, the temperature of which was controlled using a recirculating temperature controller (5750P41A10E three-quarters HP, PolyScience, IL, USA) with a temperature probe providing feedback on the temperature in the working section.

### (a) Wind tunnel air flow characteristics

Spatial variation in airspeed and axial (streamwise) turbulence intensity was measured across the central transverse plane of the working section using a hot wire anemometer (Dantec Dynamics, Skovlunde, Denmark) at three speeds (0.7, 2.58 and 4.14 m s^−1^) at equidistantly spaced horizontal and vertical positions (35 in total). The hot wire anemometer was calibrated against a Pitot tube (FCO322, Furness Controls Ltd, Bexhill-on-Sea, East Sussex, UK). Data were sampled at 1 kHz and processed using Mini-CTA software (Dantec Dynamics, Skovlunde, Denmark). A median filter was used to remove outliers in the dataset. All measurements were made with screens in place at the entrance and exit of the working section of the wind tunnel, since these screens were also in place during the bumblebee flights to restrict the bumblebee to the working section.

Turbulence intensity (*I*) was calculated as follows:


(3.1) $$I=\frac{\overset{-}{u^{\prime }}}{\overset{-}{U}},$$

where $\overset{-}{U}$ is the local time-averaged speed and $\overset{-}{u^{\prime }}$ is the root mean square fluctuation in streamwise flow speed, calculated as


(3.2) $$\overset{-}{u^{\prime }}=\sqrt{\frac{\sum {\left(u_{i}-\overset{-}{U}\right)}^{2}}{n}},$$

where $u_{i}$ is the local speed measured at time *i* and *n* is the number of samples.

Spatial variation in airspeed and turbulence intensity is illustrated in electronic supplementary material, figure S3. Across the centre of the working section where bumblebees typically flew, velocity was within −17.4 to 17.3%, −10.7 to 17.4% and −9.7 to 2.15% at 0.7, 2.58 and 4.14 m s^−1^, respectively (electronic supplementary material, figure S3). Air flow quality was least turbulent in the central portion of the working section of the tunnel, where the bumblebees typically flew (bumblebee wingspan is approximately 25 mm giving a wingspan to working section span ratio of 25%; root-mean squared axial turbulence *I*, ranged from 0.9 to 7.3% at 0.7 m s^−1^; 4.6 to 13.5% at 2.58 m s^−1^ and 1.7 to 10.4% at 4.14 m s^−1^ across the transverse plane of the working section (electronic supplementary material, figure S3). Around the edges of the working section (particularly at the highest speed) airspeed was lower than in the centre; there were several regions of higher turbulence. Turbulence intensity for some wind tunnels used for animal flight research is lower than in our wind tunnel; however, some of those measurements have been made without the upstream retaining meshes in place [17]. The presence of the screen at the entrance of the working section likely increased the level of turbulence; in the Lund wind tunnel, the inclusion of a net upstream of the working section increased axial turbulence intensity by 30× [18]. However, the level of turbulence in the wind tunnel fell well within the range bumblebees experience while foraging in the field [19].

### (b) Overall efficiency

The gross overall efficiency of flight (η) was calculated from the ratio of the mass-specific mechanical power requirement to the mass-specific gross metabolic power input ($P_{\text{i}}^{*}$). The mass-specific mechanical power requirements of flight were estimated from two studies: (i) mechanical power was calculated using a kinematic and aerodynamic analysis [20], however, the profile power was adjusted (following [21,22]) to account for the increased drag associated with the leading edge vortex (the existance of this structure was not known at the time of the original study); and (ii) mechanical power determined using a computational fluid dynamics model [23]. It is likely that there is at least some elastic storage in the bumblebee flight system [24], and therefore we used mass-specific mechanical power from both studies calculated assuming either perfect elastic energy storage ($P_{\text{o,perf}}^{*}$) or zero elastic energy storage ($P_{\text{o,zero}}^{*}$). $P_{\text{i}}^{*}$ was calculated assuming 1 ml CO_2_ produced is equivalent to 21.1 J energy [25], which is appropriate for the aerobic metabolism of carbohydrate (i.e. respiratory exchange ratio of 1.0), which has been previously determined for bumblebees [26].

### (c) Statistical analyses

Data were pooled to allow for a sufficient sample size for statistical analysis. To determine whether ${\overset{˙}{V}}_{{\text{CO}}_{2}}$ varied with flight speed, an AIC model selection was used to initially determine whether a linear, quadratic or cubic regression model best fitted these data using R (v. 4.2.0). For free flight, a cumulative model weight of 1% for linear, 72% for quadratic and 27% for cubic regression. A cumulative model weight of 2% for linear, 75% for quadratic and 23% for cubic regression was seen for tethered flight. Quadratic regression therefore was the best-fit model. Minimum power speed (*U*_mp_) and maximum range speed (*U*_mr_) were determined from the quadratic regression. *U*_mp_ was measured as the minimum of the metabolic power–speed relationship. *U*_mr_ was calculated using the equation of the tangent to the quadratic that passes through the origin. ANCOVA was used to test whether there were differences in the absolute metabolic rate or its trajectory with forward airspeed between free-flight and tethered-flight groups. ANCOVA was selected over linear mixed model (LMM) as the main aim was to investigate group-level differences in metabolic rate rather than individual trajectories. A sensitivity analysis using LMM was used to check the results were consistent. An independent *t*‐test was used to assess the effects of isoflurane by comparing ${\dot{V}}_{{\text{CO}}_{\text{2}}}^{*}$ during free flight in unanaesthetized ((${\overset{˙}{V}}_{{\text{CO}}_{\text{2, free}}}^{*}$) and following isoflurane exposure (${\overset{˙}{V}}_{{\text{CO}}_{\text{2,iso}}}^{*}$). Statistical tests were completed in SPSS (v. 26, IDM, USA) and IgorPro (v. 8.03, WaveMetrics Inc., OR, USA), and data are presented as mean ± s.d. A *p-*value < 0.05 was considered significant.

## 4. Results

### (a) Flight energetics during free and tethered flight

**Figure 1.**
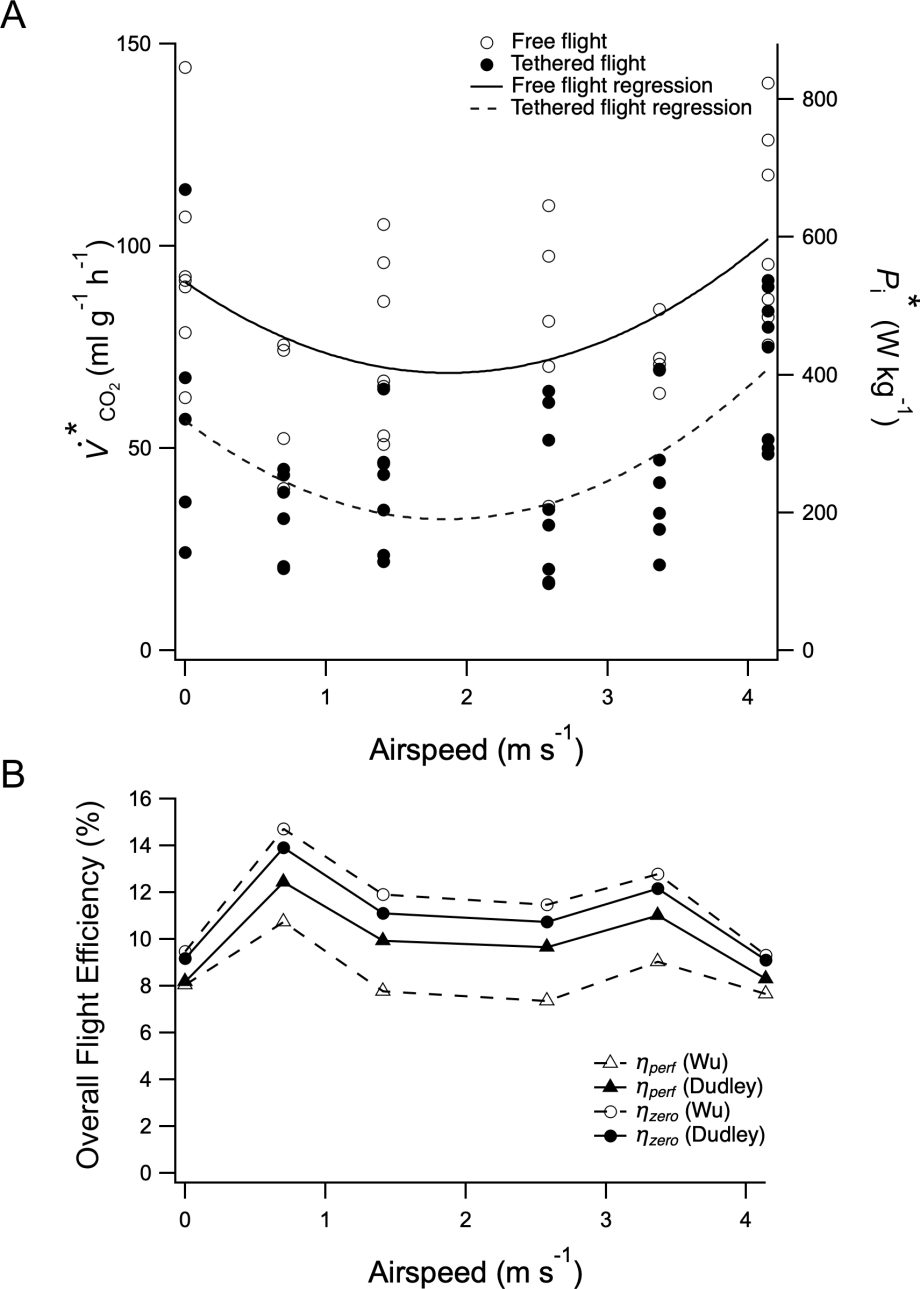
(A) Relationship between ${\dot{V}}_{{\text{CO}}_{\text{2}}}^{*}$ during hovering and forward flight for free (open circles; *n* = 7) and tethered flight (filled circles; *n* = 8) and airspeed (*U*). A significant quadratic regression model has been fitted to the free (solid line; *p* = 0.009; ${\dot{V}}_{{\text{CO}}_{\text{2}}}^{*}=6.43U^{2}-24.03U+91.10$) and tethered flight (dashed line; *p* < 0.001; ${\dot{V}}_{{\text{CO}}_{\text{2}}}^{*}=7.11U^{2}-26.31U+56.79$) data. Each marker represents a data point from an individual. (B) Overall flight efficiency (η) was calculated as the ratio of $P_{\text{o,perf}}^{*}$ or $P_{\text{o,zero}}^{*}$ ([20]; ‘Dudley’; closed symbols, solid line; and [23]; ‘Wu’; open symbols, dashed line) to our $P_{\text{i}}^{*}$, for perfect (triangular symbol) and zero elastic energy storage (circular symbol). The number of individuals studied at 0, 0.7, 1.41, 2.58, 3.37 and 4.14 m s^−1^ are: free—7, 4, 7, 5, 4, 7 and tethered—5, 6, 8, 8, 8, 7, 8, respectively.

There was a U-shaped relationship between ${\dot{V}}_{{\text{CO}}_{\text{2}}}^{*}$and airspeed during free flight (*R*^2^ = 0.224, *F*(2, 31) = 4.47, *p* = 0.009; figure 1A) and tethered flight (*R*^2^ = 0.818, *F*(2, 38) = 8.86, *p* < 0.001; figure 1A). ${\dot{V}}_{{\text{CO}}_{\text{2}}}^{*}$ ranged from 62.4 to 144.1 ml g^−1^ h^−1^, corresponding to $P_{\text{i}}^{*}$ 366.5−846.0 W kg^−1^ during hovering and 35.6−110.0ml g^−1^ h^−1^; 208.9−645.5 W kg^−1^ at 2.58 m s^−1^ (figure 1A). Across the speed range ($\in \left|0,\;4.14\;\text{m}{\text{s}}^{\text{-1}}\right|$) ${\dot{V}}_{{\text{CO}}_{\text{2}}}^{*}$ was 45.0% lower during tethered flight compared to free flight (*F*(1,69) = 9.661, *p* < 0.001, *η*^2^ = 0.12; figure 1A) and 52.7% lower at *U*_mp_. There was no significant interaction between condition and speed (*F*(1,69) = 0.031, *p* = 0.86, *η*^2^ = 0.00) or condition and speed squared (*F*(1,69) = 0.054, *p* = 0.82, *η*^2^ = 0.00), indicating that the shape of the ${\dot{V}}_{{\text{CO}}_{\text{2}}}^{*}$–speed relationship did not differ between free and tethered flight. However, a significant main effect of condition was found (*p* = 0.003) demonstrating that ${\dot{V}}_{{\text{CO}}_{\text{2}}}^{*}$ was consistently higher during free flight (figure 1A). This remained similar under LMM predictions (condition and speed *F*(1,42.21) = 0.029, *p* = 0.87; condition and speed squared *F*(1, 46.61) = 0.044, *p* = 0.84; condition *F*(1,24.51) = 6.33, *p* = 0.19).

*U*_mp_ in free flight occurred at 1.87 m s^−1^ and at 1.85 m s^−1^ during tethered flight. *U*_mr_ for free flight was 3.77 and for tethered flight was 2.83 m s^−1^.

Overall flight efficiency during hovering was in the range 8.1–8.2% (assuming perfect elastic energy storage, $\eta_{\text{perf}}$) and 9.2–9.5% (assuming zero elastic energy storage, $\eta_{\text{zero}}$), and during forward flight was between 7.3–12.4% ($\eta_{\text{perf}}$) and 9.1–14.7% ($\eta_{\text{zero}}$), but no significant relationship with airspeed was found (figure 1B; *p* > 0.05).

### (b) Effects of isoflurane on free-flight energetics

**Figure 2.**
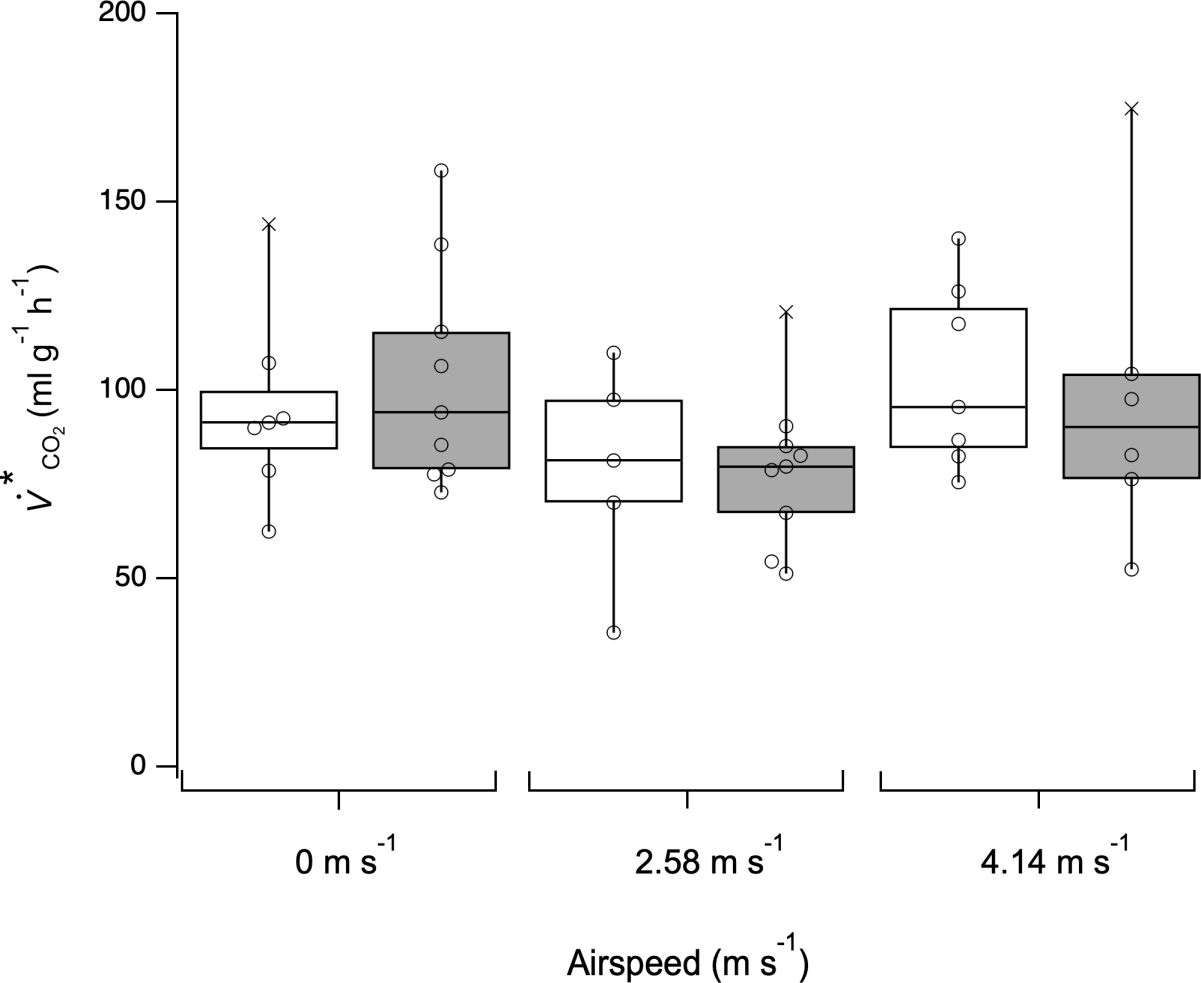
Isoflurane anaesthesia had no effect on ${\dot{V}}_{{\text{CO}}_{\text{2}}}^{*}$ during free hovering and forward flights. Control, unanaesthetized bees are represented by open boxes (*n* = 7) and post-isoflurane bees by filled boxes (*n* = 9). Solid horizontal line represents median ${\dot{V}}_{{\text{CO}}_{\text{2}}}^{*}$. Box represents first and third quartiles. Bars represent the maximum and minimum ${\dot{V}}_{{\text{CO}}_{\text{2}}}^{*}.$

There was no significant effect of isoflurane on free-flight energetics at airspeeds 0 m s^−1^ (*t*(14) = −0.563, *p* = 0.582), 2.58 m s^−1^ (*t*(12) = 0.499, *p* = 0.998) and 4.14 m s^−1^ (*t*(11) = 0.388, *p* = 0.777), compared to controls (figure 2).

## 5. Discussion

### (a) Free-flight energetics

There was a U-shaped relationship between gross mass-specific metabolic power and flight speed during free flight across the speed range. Our data therefore provide support for a speed-dependent power–speed relationship, previously only quantified in tethered locusts and honeybees [12,13] and in free-flying drone flies [9]. However, the U-shaped metabolic power–speed relationship contrasts with the only other experimental analysis of free-flight energetics in bumblebees*,* where oxygen consumption was independent of forward flight speed [10]. Why a different relationship was observed is unclear. Compared to previously published studies, ${\overset{˙}{V}}_{{\text{CO}}_{2}}$ in this study during hovering appears to be considerably higher. For hovering, average $P_{\text{i}}^{*}$ in this study was 559 W kg^−1^, whereas in previously published reports hovering metabolic rate has typically ranged from 410 to 435 W kg^−1^ [10,27]. There was still overlap in ${\dot{V}}_{{\text{CO}}_{\text{2}}}^{*}$ between individuals for this study and the published hovering literature; however, as the U-shaped power curve observed in this study is highly dependent on the hovering and low-speed flights, it is important to establish the possible influences on hovering metabolic rate. It is likely, our stricter flight behaviour characterization that excluded buzzing and walking behaviours which were included in the ‘flight’ trials of Ellington *et al*.’s [10] study, may have better resolved the metabolic power requirements.

The shallow U-shaped metabolic power–speed relationship observed in this study is, however, consistent with shallow U-shaped curves that were seen for some individual bees in Ellington *et al*.’s study [10,11]. Additionally, the current study also used a single worker caste of a single species, which may have reduced the confounding effects of body mass, caste-specific and inter-specific anatomical and physiological differences not accounted for in Ellington *et al*.’s [10] study. A significant U-shaped relationship was still observed when these free-flight data were combined with a separate free-flight study group (figure 1A; electronic supplementary material, figure S4). Although the average hovering ${\dot{V}}_{{\text{CO}}_{\text{2}}}^{*}$ was lower, for the combined groups, there was, importantly, still no significant difference across the speed range between the groups or the trajectories of their metabolic power–speed relationship between free and tethered flight (figure 1A; electronic supplementary material, figure S4). The U-shaped relationship reported here for ${\dot{V}}_{{\text{CO}}_{\text{2, free}}}^{*}$, therefore indicates that both flight speed and metabolic cost should be considered when attempting to interpret bumblebee behaviour, and that the assumption that metabolic rate for hovering flight can be used as a proxy for all flight speeds [10] is erroneous.

An aerodynamic analysis of bumblebee flight determined a shallow U-shaped relationship between mechanical power and speed across a similar range of speeds [20], but was carried out before the existence of the leading-edge vortex in insect flight was known [28], and therefore ignored the increase in profile power associated with this phenomenon [21]. More recent estimates of the mechanical power–speed relationship based on unsteady aerodynamic forces are either U- or J-shaped relationship, depending on assumptions about elastic energy storage [23]. Limited measurements of the overall efficiency of flight, and whether this varies with speed, have led to uncertainty in whether the shapes of the metabolic and mechanical power–speed relationships should match. Here, we demonstrate that the overall efficiency of flight does not vary significantly with airspeed, and therefore the shape of the metabolic power–speed curve can be approximated from the mechanical power–speed curve. However, in estimating metabolic power from mechanical power, body mass must also be considered due to the scaling of overall efficiency [22]. The overall efficiency determined here from these metabolic data and mechanical power estimates from [20,23] gave an overall efficiency range of 7.3–14.7%, depending on assumptions about elastic energy storage. This coincides with earlier estimates using bumblebee flight muscle power output (2.5%; [29]) and hovering free flight (16.9%; [22]). Direct measurements of insect flight muscle *net* efficiency are slightly higher (beetle 14–16%; [24]) than our estimates of *gross* overall efficiency. This is expected for several reasons: (i) the latter includes the resting metabolic rate; (ii) there may be inefficiencies in the transfer of muscle mechanical to aerodynamic work; and (iii) the beetles had a lower wingbeat frequency than bumblebees and are therefore expected to have a higher overall efficiency [22].

### (b) Energetics of tethered flight compared to free flight

A direct comparison of metabolic rate during free flight and tethered flight demonstrated a 45% reduction in ${\dot{V}}_{{\text{CO}}_{\text{2}}}^{*}$, on average, during tethered flight (34.97% reduction in combined free-flight dataset; electronic supplementary material, figure S4) of a magnitude slightly lower than that previously reported in forward flight: e.g. 56% in tobacco hawkmoth (flying at 1.73 m s^−1^ [14]) and 72% in mango stem borer (flying at 3.5 m s^−1^ [16]). The reduction in metabolic rate is unsurprising, given that the animal does not need to support its body weight, and therefore lift production is likely to be lower than in free flight [30]. This is the likely explanation for the reduction in wingbeat frequency in tethered insects [31], which is expected to be associated with a reduction in metabolic rate [2,32,33].

Despite the difference in the magnitude of metabolic power at any given speed, there was little variation in *U*_mp_ between free (1.87 m s^−1^) and tethered flight (1.85 m s^−1^), indicating that tethered-flight energetics (at least in bumblebees) can be used as a proxy for the minimum metabolic power during free flight, in instances where free-flight measurements are difficult. However, there was a larger difference in *U*_mr_ between tethered and free flight, with *U*_mr_ being underestimated by approximately 25% in tethered flight. Therefore, predictions about animal behaviour based on *U*_mr_ obtained during tethered flight need to be made cautiously.

### (c) Isoflurane anaesthesia as an approach to immobilizing insects for energetics studies

It is important that methods used to immobilize insects for experimental investigation should not adversely influence traits subsequently studied. Insect immobilization via CO_2_ or cold exposure can have a significant physiological and behavioural effects [34,35]. CO_2_ exposure disrupts metabolic regulation in insects and should only be used with caution in physiological studies [36]. Immobilization by cold exposure can take an unpredictable time in facultative endotherms, e.g. bumblebees. Developing an alternative immobilization method is thus advantageous. Here, we demonstrated that isoflurane anaesthesia had no lasting effect on flight energetics (figure 2), supporting the use of this method as a means of immobilization, though sufficient recovery time must be allowed before experimental use of insects since metabolic rate is still reduced during the recovery period [37].

## Data Availability

Bumblebee metabolic rate (mass specific rate of carbon dioxide production) in relation to airspeed for free flight, tethered flight and free flight following exposure to isoflurane is available at: [38]. Supplementary material is available online [39].
